# Interpretable machine learning with Bayesian optimization for bond strength prediction of steel reinforcement in geopolymer concrete

**DOI:** 10.1371/journal.pone.0352645

**Published:** 2026-07-09

**Authors:** Viet Hung Tran, Viet Hai Hoang, Quang Minh Tran

**Affiliations:** 1 Faculty of Civil Engineering, University of Transport and Communications, Hanoi, Vietnam; 2 Department of Civil Engineering, University of Minho, Guimarães, Portugal; Instituto Federal do Espírito Santo: Instituto Federal de Educacao Ciencia e Tecnologia do Espirito Santo, BRAZIL

## Abstract

Accurate estimation of bond strength between steel reinforcement and geopolymer concrete is essential for the reliable design of sustainable reinforced concrete structures. However, the highly nonlinear interactions reduce the applicability and accuracy of conventional empirical models. This study proposes a Bayesian-optimized interpretable machine learning framework to predict the ultimate bond strength of reinforced geopolymer concrete using a comprehensive experimental database compiled from published studies. A dataset of 238 samples with 20 influential input variables was assembled to represent material properties, geopolymer chemistry, and specimen geometry. Six advanced machine learning algorithms, including Support Vector Regression (SVR), Random Forest (RF), Extra Trees Regressor (ETR), Gradient Boosting Machine (GBM), XGBoost, and CatBoost, were developed and systematically compared. Hyperparameter tuning was performed using Bayesian optimization to improve model performance. The results indicate that all models achieved strong predictive capability, while the optimized CatBoost model (BO-CatBoost) provided the best performance with testing metrics of R² = 0.950, MAE = 1.173, MAPE = 11.608%, and RMSE = 1.669. A comparative evaluation with existing empirical equations further demonstrated the superior accuracy and lower prediction variability of the proposed model. To enhance model transparency, SHAP-based explainability analysis was conducted to quantify the contribution of each input parameter. The global importance analysis revealed that compressive strength, the embedment length-to-bar diameter ratio, and the cover-to-bar diameter ratio are the most influential factors governing bond strength. Additional mixture-related parameters, including the alkaline solution-to-binder ratio, curing temperature, CaO content in the binder, and the SiO₂/Al₂O₃ ratio, also contribute to the bond mechanism by influencing geopolymerization and matrix densification. The proposed framework provides both high predictive accuracy and interpretable insights, demonstrating the potential of Bayesian-optimized interpretable machine learning to support the design and optimization of sustainable reinforced geopolymer concrete structures.

## 1. Introduction

The construction industry is a major source of global carbon dioxide emissions. This impact is largely associated with the widespread use of ordinary Portland cement (OPC) in conventional concrete. The production of cement alone contributes approximately 7–8% of global CO₂ emissions. This concern has driven increasing interest in sustainable alternatives to traditional cementitious materials. Among these, geopolymer concrete has received considerable attention. It is formed through the alkaline activation of aluminosilicate materials, such as fly ash and ground granulated blast-furnace slag. Geopolymer concrete offers several advantages over OPC-based concrete. These advantages include reduced carbon emissions, high early strength, excellent durability, and improved resistance to chemical attack [1–5]. As a result, geopolymer concrete has been increasingly investigated as a sustainable construction material [6].

The bond between steel reinforcement and concrete is a key factor in the performance of reinforced concrete structures. It governs composite action, stress transfer, crack control, and structural integrity [7]. This behavior is governed by multiple interacting parameters, including material properties, curing conditions, and geometric characteristics. Existing empirical equations remain limited in applicability because they are often developed for specific test conditions and do not fully capture the influence of geopolymer composition. Sufficient bond strength ensures the effective transfer of stresses from reinforcing bars to the surrounding concrete through adhesion, friction, and mechanical interlocking [8]. Experimental studies have reported that the bond behavior of geopolymer concrete differs from that of conventional OPC concrete because of differences in microstructure, chemical composition, and interface characteristics [9,10]. Moreover, bond performance is strongly influenced by several parameters, including compressive strength, embedment length, bar diameter, concrete cover, and curing conditions [10,11].

Recent studies on advanced cementitious composites, particularly ultra-high-performance concrete (UHPC) [12], have demonstrated that interfacial bond behavior is strongly associated with microstructural characteristics, binder composition, and matrix densification [13]. In repair and strengthening applications of reinforced concrete structures, UHPC jacketing systems have demonstrated enhanced bond performance and stress-transfer capacity, driven by improved interfacial transition zones (ITZs) and superior mechanical interlocking [7,14]. These findings suggest that the bond behavior of cementitious composites cannot be adequately characterized solely by compressive strength and geometric parameters, especially when the binder system differs fundamentally from conventional OPC concrete. Similar challenges are expected in geopolymer concrete systems, where the alkaline activation process, Si/Al ratio, activator composition, and curing regime may significantly influence the steel–concrete interface behavior [15,16].

Several empirical and analytical models have been proposed to estimate bond strength in conventional reinforced concrete systems. Design codes and classical models, such as those developed by Orangun [8], and American Concrete Institute, were originally calibrated using experimental data obtained from OPC concrete structures. Consequently, these models may not accurately predict the bond strength of geopolymer concrete due to its distinct mechanical and chemical properties [8]. Recent experimental investigations have reported noticeable deviations when conventional bond models are applied to geopolymer concrete systems [17]. To address this issue, several researchers have attempted to develop empirical equations specifically for geopolymer concrete. For instance, Topark-Ngarm [11] and Dahou [18] proposed a predictive equation for the bond strength between steel reinforcement and geopolymer concrete based on the compressive strength of the material. Similarly, Kim and Park [19] suggested bond strength equations for geopolymer concrete that are structurally similar to the formulation proposed by Orangun [8]. However, these equations primarily rely on compressive strength and geometric parameters, and they do not explicitly consider the influence of geopolymer mixture composition on bond behavior. Despite the increasing research interest in geopolymer concrete, several important challenges remain in understanding and predicting the bond behavior between steel reinforcement and geopolymer concrete. The current limitations can be summarized as follows:

i Research on the bond behavior between steel reinforcement and geopolymer concrete remains relatively limited.
ii Most existing findings are based on individual experimental studies with restricted testing ranges.
iii The bond response is governed by multiple coupled factors, including material composition, curing regimen, and geometric characteristics.
iv Variations in testing procedures, specimen configurations, and data reporting reduce the consistency and comparability of published results.
v Current empirical formulations have limited generalizability, underscoring the need for robust, data-driven predictive models.

To overcome the limitations of traditional empirical equations, machine learning (ML) techniques have recently been introduced in civil engineering to predict complex material behaviour. ML algorithms can capture complex nonlinear relationships between input variables and structural performance [20]. This capability makes ML well-suited for modeling the properties of concrete materials. In recent years, various ML techniques have been applied to geopolymer concrete. These techniques include Random Forest, Extreme Gradient Boosting, and CatBoost [21]. Previous studies have used these models to predict important mechanical properties, such as compressive strength, tensile strength, and durability-related parameters [22,23]. The results indicate that ML-based models often achieve higher predictive accuracy than conventional regression methods.

Despite these promising developments, important challenges remain in applying machine learning to bond strength prediction. The first challenge is hyperparameter selection. The performance of machine learning models is highly sensitive to hyperparameter choices. Poorly selected values may reduce prediction accuracy, leading to overfitting or underfitting. For this reason, efficient hyperparameter optimization is necessary. Bayesian optimization has recently emerged as a powerful approach for this purpose. It provides a systematic and automated search strategy while maintaining low computational cost [24]. The second challenge is model interpretability. Many accurate machine learning models function as black boxes. Their internal decision-making processes are often difficult to explain. Consequently, the contribution of each input variable to the predicted bond strength may remain unclear. This lack of transparency restricts the practical use of machine learning in structural engineering, where physical interpretation and engineering confidence are essential.

Interpretable machine learning techniques have been increasingly adopted to improve the transparency of model predictions. These methods provide clearer insight into how machine learning models generate their outputs. Among them, SHAP and Permutation Feature Importance are widely used to quantify the contribution of individual input variables [25]. Such techniques improve the interpretability and credibility of ML-based predictions. In bond strength studies, they also help identify the key factors governing the interaction between steel reinforcement and geopolymer concrete.

The present study proposes a Bayesian-optimized interpretable machine learning framework for predicting the bond strength between steel reinforcement and geopolymer concrete. The main objectives of this study are: (i) to develop machine learning models capable of accurately predicting bond strength based on experimental datasets; (ii) to optimize the hyperparameters of the developed models using Bayesian optimization to enhance prediction performance; and (iii) to interpret the trained models using explainable artificial intelligence techniques in order to identify the most influential parameters governing bond behavior.

The main contributions of this research can be summarized as follows:

1. To compile and analyze an experimental database of bond strength between steel reinforcement and geopolymer concrete collected from published literature.
2. To develop several machine learning models capable of predicting bond strength based on key influencing parameters, including geometric characteristics, mechanical properties, and mixture-related variables.
3. To apply Bayesian optimization techniques to systematically tune the hyperparameters of the developed machine learning models in order to improve prediction accuracy and model robustness.
4. To employ interpretable machine learning methods, such as SHAP, to quantify the relative importance of input variables and reveal the underlying relationships governing bond behavior.
5. To compare the predictive performance of the proposed machine learning models with existing empirical equations and design provisions for bond strength prediction.

By integrating predictive accuracy with model interpretability, this study provides a robust framework for estimating the bond strength between steel reinforcement and geopolymer concrete. The findings contribute to a better understanding of the factors governing bond behavior and offer a useful basis for the development of reliable prediction tools for sustainable reinforced concrete design. The remainder of this paper is organized as follows. Section 2 presents the methodological background, including database construction, machine learning models, Bayesian optimization, and SHAP-based interpretation. Section 3 describes model implementation and evaluates predictive performance. Section 4 provides the explainability analysis of the selected model. Section 5 discusses the limitations of the present study and offers directions for future research. Section 6 concludes the paper.

## 2. Methodological background

### 2.1. Data construction from experimental database

**Fig 1:**
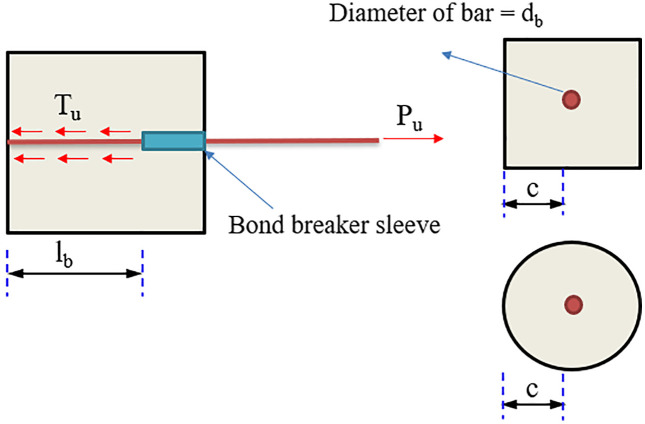
Schematic illustration of the bond between steel reinforcement and geopolymer concrete in the pull-out test.

In this study, a database containing 238 experimental data points was established to investigate the bond behavior between steel reinforcement and geopolymer concrete (Fig 1). The collected data were used to develop and evaluate predictive models of bond strength. The database was assembled from 23 peer-reviewed studies reporting laboratory tests on the bond performance of steel bars embedded in geopolymer concrete [2,10,11,15,17,18,26–41] and [42], as summarized in Table 2. Most of the specimens were tested using standard pull-out tests. A smaller number of studies adopted beam-end or lap-splice test configurations (Table 1).

**Table 1:** Statistical information for parameters in the databases.

No	Feature	Type	Units	Min	Max	Mean	Median	Std	S	K
1	FA	X1	kg/m^3^	80	525.30	400.94	408.00	72.53	−1.80	5.45
2	Slag	X2	kg/m^3^	0	240.00	18.04	0.00	51.60	2.98	8.16
3	SiO_2_/Al_2_O_3_	X3	–	1.20	5.08	2.26	2.07	0.81	1.84	3.74
4	CaO	X4	%	0.40	33.81	6.53	3.98	6.85	1.81	3.24
5	CAgg	X5	kg/m^3^	912.77	1411.00	1147.91	1172.62	102.36	−0.16	−0.18
6	FAgg	X6	kg/m^3^	471.00	893.00	626.57	630.00	77.33	0.66	1.68
7	NaOH (M)	X7	mol	8	20	13.11	14.00	2.76	−0.15	0.47
8	Na_2_SiO_3_/NaOH	X8	–	0	7.94	2.29	2.50	1.08	2.73	11.33
9	AS/B	X9	–	0.265	0.75	0.48	0.48	0.13	0.38	−1.04
10	W/B	X10	–	0.067	0.30	0.16	0.13	0.07	0.83	−0.68
11	Sf	X11	Kg/m3	0	22.50	2.10	0.00	3.30	1.80	5.30
12	T^o^	X12	^0^C	0	90	40.17	60.00	36.72	−0.08	−1.79
13	Duration	X13	hours	0	168	14.12	24.00	18.57	4.75	38.06
14	Age	X14	Day	1	180	33.89	28.00	35.99	2.72	8.03
15	fc’	X15	MPa	16	73.41	37.27	35.89	12.19	0.52	0.00
16	d_b_	X16	mm	8	25.00	16.43	16.00	3.46	0.79	0.70
17	f_y_	X17	MPa	305.09	649.88	502.53	500.00	60.74	−0.61	0.46
18	c/d_b_	X18	–	1.04	7.83	4.00	4.19	1.48	0.25	−0.10
19	l_b_/d_b_	X19	–	2.00	30.13	6.33	5.00	3.49	3.85	19.17
20	τ_u_	Y	MPa	2.49	32.00	16.61	14.60	7.59	0.29	−1.02

Note: Min. = minimum; Max. = maximum; Mean = average; Median = median value; Std. = standard deviation; S = Skewness; K = Kurtosis.

**Table 2:** Optimal Hyperparameters of Six Machine Learning Models.

Model	Hyperparameter range		Optimal value	Model	Hyperparameter range		Optimal value
SVR	kernel	“rbf,” “poly,” “sigmoid”	rbf	GBM	n_estimators	[100-1000]	406
					learning_rate	[0.01-0.2]	0.0451
	C	[0.1, 1000.0]	35.208		max_depth	[3–10]	4
	gamma	Scale, Auto	Scale		min_samples_split	[10-30]	11
	epsilon	[0.01-1.0]	0.473		min_samples_leaf	[1-20]	8
					subsample	[0.6-1.0]	0.7770
ETR	n_estimators	[100-1000]	517	XGBoost	learning_rate	[0.005-0.02]	0.0172
	max_depth	[5-30]	16		max_depth	[2–6]	4
	min_samples_split	[2-20]	6		min_child_weight	[5,20]	5
	min_samples_leaf	[1–10]	1		subsample	[0.6,0.8]	0.7126
	max_features	[0.5-1]	0.574		colsample_bytree	[0.6,0.8]	0.7164
	bootstrap	True, False	False		gamma	[0-5]	0.1247
					reg_alpha	[0-10]	0.0503
					reg_lambda	[0-20]	7.23
RF	n_estimators	[100-1000]	0.0296	CatBoost	learning_rate	[0.005-0.05]	0.0483
	max_depth	[5-30]	6		max_depth	[2–6]	6
	min_samples_split	[2-20]	11.142		l2_leaf_reg	[10-50]	15.453
	min_samples_leaf	[1–10]	7.663		random_strength	[1–10]	6.052
	max_features	[0.5-1]	0.635		bagging_temperature	[0.5-1.0]	0.607

The data collection process included numerical values reported in experimental tables, parameter descriptions provided in the text, and values digitized from published figures when tabulated data were not directly available. After data collection, the compiled database was organized into input and output variables for subsequent modeling. Because the database was compiled from multiple independent literature sources, a data consistency-control procedure was conducted before model development. All extracted data were first checked for consistency in variable definitions, units, and admissible value ranges. Reported values were converted into common units when necessary. Derived geometric variables were then recalculated when sufficient original information was available. These variables included the concrete cover-to-bar diameter ratio (c/d_b_) and the embedment length-to-bar diameter ratio (l_b_/d_b_). Duplicated records were removed from the database. Incomplete records were also excluded when essential variables were missing. Major sources of experimental variability were retained as input variables in the machine learning models. These sources included mixture composition, curing temperature, curing duration, testing age, bar diameter, concrete cover, embedment length, and bar type. This procedure improved data consistency. It also allowed the models to account for inter-study differences in materials, curing conditions, and geometric conditions. The final dataset contains 19 numerical input variables describing material properties, geopolymer mixture composition, specimen geometry, and reinforcement characteristics. One additional categorical input variable was used to identify the bar type, namely ribbed or plain.

The selected input variables were chosen to represent the main physical, chemical, and geometric factors governing the bond behavior between steel reinforcement and geopolymer concrete. These variables can be grouped into four categories: binder and mixture composition, activator characteristics, curing conditions, and reinforcement/specimen geometry. Binder-related variables, such as fly ash content, slag content, CaO content, and the SiO_2_/Al_2_O_3_ ratio, were included because they influence geopolymerization, reaction products, matrix density, and mechanical strength development. Activator-related variables, including NaOH concentration, Na_2_SiO_3_/NaOH ratio, alkaline solution-to-binder ratio, and water-to-binder ratio, were considered because they affect dissolution of aluminosilicate precursors, reaction kinetics, workability, porosity, and microstructural formation. Curing-related variables were included because temperature, duration, and age control the extent of reaction and the degree of strength gain. Mechanical and geometric variables, such as compressive strength, bar diameter, reinforcement yield strength, concrete cover-to-bar diameter ratio, and embedment length-to-bar diameter ratio, were included because they directly govern adhesion, friction, mechanical interlock, confinement, and stress transfer along the steel–concrete interface.

**Fig 2:**
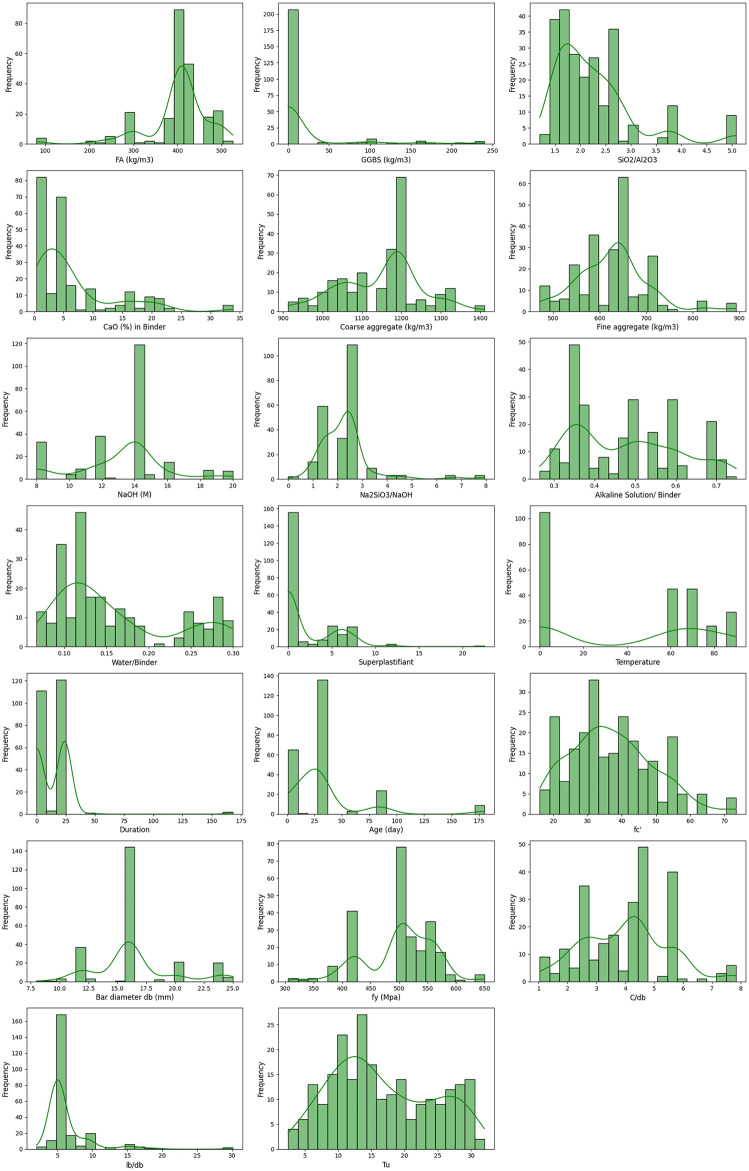
Distribution of 19 numerical input and 01 numerical output features.

To incorporate this categorical variable into the machine learning models, a one-hot encoding technique was employed, whereby the bar type was transformed into binary indicator variables representing the two categories. This encoding approach enables the learning algorithms to properly interpret categorical information without introducing artificial ordinal relationships between categories. The output variable corresponds to the bond strength between steel reinforcement and geopolymer concrete. To characterize the dataset, descriptive statistics were computed for all input and output variables. As presented in Table 1 and Fig 2, the statistical summary includes the minimum, maximum, mean, median, standard deviation, skewness, and kurtosis of each variable. These indicators were used to examine the distribution, spread, and heterogeneity of the collected data before model development. The statistical results show that the database covers a broad range of geopolymer concrete mixtures and test conditions, which is important for developing predictive models that are adequately representative.

The descriptive statistics also indicate substantial variability in several key parameters. The fly ash content (FA) varies from 80 to 525.30 kg/m³, with an average value of 400.94 kg/m³. This indicates that most mixtures are predominantly fly ash–based. In contrast, the ground granulated blast furnace slag (Slag) content ranges from 0 to 240 kg/m³ with a highly skewed distribution (skewness = 2.98). The slag was incorporated only in a limited portion of the mixtures.

The chemical composition of the binder shows substantial variation across the collected studies. The SiO_2_/Al_2_O_3_ ratio ranges from 1.20 to 5.08, with a mean value of 2.26. The CaO content ranges from 0.40% to 33.81%. These results reflect the diversity of precursor materials used in geopolymer concrete. Binder chemistry plays a key role in the geopolymerization process. It also affects the resulting microstructure and bond behavior of the material.

The proportions of the mixture also vary widely. The coarse aggregate content ranges from 912.77 to 1411 kg/m³. The fine aggregate content ranges from 471 to 893 kg/m³. The activator-related parameters also show significant variability. The sodium hydroxide concentration ranges from 8 to 20 M. The sodium silicate-to-sodium hydroxide ratio ranges from 0 to 7.94 and exhibits strong right skewness (skewness = 2.73). The alkaline solution-to-binder ratio ranges from 0.265 to 0.75. The water-to-binder ratio ranges from 0.067 to 0.30. These parameters strongly influence the reaction kinetics of the geopolymer system. They also affect the resulting mechanical properties of geopolymer concrete.

The curing conditions included in the database are also diverse. The curing temperature (T^o^) ranges from 0 to 90°C with a mean of 40.17°C, while the curing duration varies substantially from 0 to 168 hours, showing extremely high skewness (skewness = 4.75), indicating that most specimens were cured for relatively short periods with a few cases of prolonged curing. The testing age ranges from 1 to 180 days, reflecting both early-age and long-term bond behavior.

Regarding mechanical and geometric parameters influencing bond performance, the compressive strength of geopolymer concrete (fc’) ranges from 16 to 73.41 MPa with a mean value of 37.27 MPa. The bar diameter (d_b_) varies from 8 to 25 mm, while the yield strength of reinforcement (f_y_) ranges between 305.09 and 649.88 MPa. Additionally, the concrete cover-to-bar diameter ratio (c/d_b_) spans from 1.04 to 7.83, and the embedment length-to-bar diameter ratio (l_b_/d_b_) varies widely from 2.0 to 30.13, with strong positive skewness (3.85). These geometric parameters are well known to significantly affect the bond stress transfer mechanisms between steel reinforcement and surrounding concrete.

The target variable, namely the ultimate bond-related load capacity (*τ*_*u*_), ranges from 2.49 to 32.0 MPa with an average value of 16.61 MPa and moderate variability (standard deviation = 7.59 MPa). The relatively wide distribution of this parameter reflects the significant influence of mixture composition, curing conditions, and reinforcement detailing on the bond performance of geopolymer concrete.

The statistical characteristics reveal substantial variability and non-normal distributions across several key parameters. Such complexity highlights the limitations of conventional empirical formulations and motivates the application of advanced machine learning techniques capable of capturing nonlinear relationships and complex interactions among material composition, curing conditions, and reinforcement parameters governing the bond behavior between steel reinforcement and geopolymer concrete.

**Fig 3:**
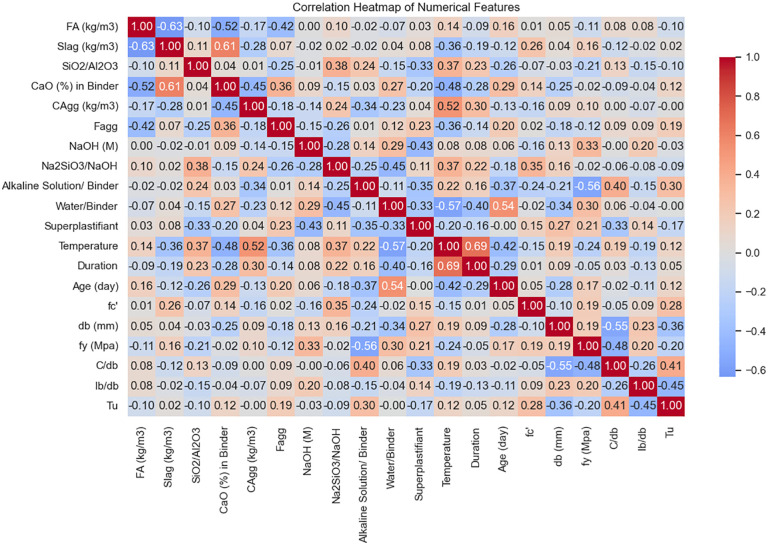
Correlation matrix for the numerical input parameters of the dataset.

Fig 3 presents the correlation matrix of the numerical input variables included in the database. A preliminary overview of the linear relationships among the parameters under consideration is provided. The observed correlation patterns indicate that the input variables exhibit different levels of association rather than a uniform trend. This result suggests that bond behavior is governed by multiple interacting factors, including material composition, curing conditions, and specimen geometry. The use of machine learning approaches can capture the complex relationships among the variables more effectively.

### 2.2. Machine learning models

This section introduces the machine learning models employed in the present study. These models include Support Vector Regression (SVR), Random Forest (RF), Extremely Randomized Trees (ETR), Gradient Boosting Machine (GBM), XGBoost, and CatBoost.

These algorithms represent both ensemble tree-based learning approaches and a kernel-based regression method. Their predictive performances were systematically evaluated and compared to identify the most reliable model for estimating the bond strength of steel reinforcement embedded in geopolymer concrete.

All machine learning models were implemented as supervised regression models, with the ultimate bond stress (τ_u_) used as the output variable. The numerical input variables were used after data preprocessing and consistency checking. The categorical variable representing bar type was converted into binary variables using one-hot encoding. For the SVR model, the input variables were standardized because kernel-based methods are sensitive to feature scale. In contrast, the tree-based ensemble models, including RF, ETR, GBM, XGBoost, and CatBoost, were trained using their original numerical scales because these algorithms are relatively insensitive to monotonic feature scaling. The SVR model was implemented using a kernel-based regression formulation. The RF and ETR models were implemented as bagging-based tree ensembles, where the final prediction was obtained by averaging the outputs of multiple decision trees. The GBM, XGBoost, and CatBoost models were implemented as boosting-based ensembles, where decision trees were sequentially constructed to improve prediction accuracy. XGBoost further included regularization, shrinkage, and subsampling mechanisms, while CatBoost used ordered boosting and symmetric trees to improve generalization and reduce overfitting. Each model was trained on the training subset and optimized using Bayesian optimization combined with 5-fold cross-validation. After identifying the optimal hyperparameters, the final model was retrained using the full training set and evaluated on the hold-out testing set. This implementation strategy ensured a consistent comparison among all machine learning models.

#### 2.2.1. Support Vector Regression (SVR).

Support Vector Regression (SVR) [43] is a supervised machine learning method widely used for nonlinear regression. It aims to determine an optimal regression function while controlling prediction error within a predefined tolerance range. Unlike conventional regression methods, SVR emphasizes generalization performance rather than only fitting the training data. This characteristic makes it suitable for problems with complex relationships between input and output variables.

SVR can model nonlinear behavior using kernel functions. Among the available kernel types, the radial basis function (RBF) kernel is commonly used for its flexibility and strong predictive performance. The performance of SVR is mainly governed by several hyperparameters. These include the penalty parameter C, the insensitive loss parameter ε, and the kernel parameter 𝛾. Proper selection of these parameters is essential for balancing model complexity and prediction accuracy. Because of its robustness and strong generalization ability, SVR is considered an appropriate model for predicting bond strength in geopolymer concrete.

#### 2.2.2. Randome Forest (FR).

Random Forest (RF) [44] is an ensemble learning algorithm based on multiple decision trees. It generates predictions by combining the outputs of multiple trees trained on different bootstrap samples of the training data. At each split, only a random subset of input variables is considered. This mechanism increases model diversity and reduces the risk of overfitting. RF is well-suited for nonlinear regression problems with complex interactions among variables. Its performance is influenced by hyperparameters such as the number of trees, the maximum tree depth, and the minimum number of samples required for splitting. Due to its robustness and stable predictive capability, RF has been widely applied in engineering prediction tasks.

For regression problems, the final prediction is obtained by averaging the outputs of all trees. The main hyperparameter influencing model performance is the number of trees (n_estimators). Increasing this value generally improves model stability, although the improvement becomes marginal beyond a certain point while computational cost increases. Therefore, an appropriate number of trees should be selected to balance predictive accuracy and efficiency.

#### 2.2.3. Extremely Randomized Trees (ETR).

Extremely Randomized Trees (ETR) [45] is an ensemble learning algorithm based on a collection of decision trees. It is designed to improve predictive accuracy and model stability by combining the outputs of multiple randomized trees. Similar to Random Forest, ETR builds many trees and aggregates their predictions to obtain the final result. However, ETR introduces a higher degree of randomness during the tree construction process. In this method, the training samples are usually used without bootstrap resampling, and the split thresholds are selected randomly for each candidate feature. This strategy distinguishes ETR from Random Forest, in which the best split is selected from a subset of features.

The additional randomness introduced in ETR helps reduce the variance of the model. It also decreases the risk of overfitting, especially when the dataset contains complex nonlinear relationships and interacting variables. Because the split points are generated randomly, the computational cost of tree construction can also be reduced. This characteristic makes ETR efficient for regression tasks involving a relatively large number of features. At the same time, the ensemble averaging process improves model robustness and generalization capability.

The predictive performance of ETR is controlled by several hyperparameters. These include the number of trees, the maximum tree depth, the minimum number of samples required to split a node, the minimum number of samples required at a leaf node, and the number of features considered at each split. Proper tuning of these parameters is important for achieving a balance between prediction accuracy and model complexity. Owing to its ability to capture nonlinear patterns, reduce variance, and maintain computational efficiency, ETR is considered a suitable model for predicting the bond strength between steel reinforcement and geopolymer concrete.

#### 2.2.4. Gradient Boosting Machine (GBM).

Gradient Boosting Machine (GBM) [46] is an ensemble learning algorithm that sequentially builds a series of decision trees, where each new tree is trained to reduce the residual errors of the previous model. By employing gradient-based optimization together with a learning rate that controls model complexity, GBM is capable of capturing nonlinear relationships and interactions among input variables. Owing to its strong predictive accuracy and flexibility, GBM has been widely applied in regression problems within engineering applications.

The bond behavior is governed by multiple interacting factors, including reinforcement characteristics, geometric parameters, and geopolymer mixture properties, which exhibit highly nonlinear relationships. By iteratively learning from residual errors and refining predictions, GBM can effectively model these complex dependencies and provide reliable estimates of bond strength, making it a robust approach for predicting steel–geopolymer bond performance.

#### 2.2.5. XGBoost.

Extreme Gradient Boosting (XGBoost) [47] is an advanced implementation of gradient boosting designed for high efficiency and strong predictive performance. It improves the conventional boosting framework through regularization, parallel computation, and optimized tree learning. These features enhance both model accuracy and computational speed. XGBoost can effectively capture complex nonlinear patterns in engineering datasets. It is particularly suitable for structured tabular data with multiple interacting variables. The model performance is influenced by several hyperparameters, including the number of trees, the learning rate, the maximum tree depth, and the regularization terms. Because of its robustness and flexibility, XGBoost has become one of the most widely used machine learning algorithms in predictive modeling.

The prediction of bond strength in steel-reinforced geopolymer concrete involves multiple variables. These variables are related to material properties, specimen geometry, and reinforcement characteristics. Their combined effects are complex and highly nonlinear. XGBoost is well suited for this type of prediction problem. It combines strong predictive capability with an efficient and regularized learning framework. These features allow the model to capture complex relationships in the dataset and generate reliable estimates of bond strength [48].

#### 2.2.6 Catboost.

CatBoost is a gradient boosting algorithm that is designed to improve predictive accuracy and model generalization [49]. It belongs to the family of boosting-based ensemble methods. The algorithm constructs a sequence of decision trees, each of which is developed to reduce the prediction error of the preceding ensemble. Through this iterative learning process, CatBoost can progressively improve model performance and capture complex relationships between input variables and output responses. A key advantage of CatBoost is its ability to reduce overfitting during training. This is achieved through ordered boosting, which improves learning and reduces the prediction bias that can arise in conventional boosting algorithms. CatBoost also employs symmetric tree structures. These trees improve computational efficiency and contribute to model stability. As a result, CatBoost often shows strong predictive performance, especially for structured tabular datasets.

CatBoost is also known for its capability to handle categorical features effectively. Although the present study contains only one categorical input variable, namely the bar type, this characteristic remains beneficial for the modeling framework. In addition, CatBoost performs well on datasets with nonlinear interactions among variables. This is particularly important for bond-strength prediction, because bond response is governed by multiple coupled factors, including material properties, geopolymer mixture composition, curing conditions, and specimen geometry. The predictive performance of CatBoost depends on several hyperparameters. These include the number of boosting iterations, learning rate, tree depth, and regularization settings. Proper tuning of these parameters is necessary to achieve a balance between model complexity, training stability, and prediction accuracy. Owing to its strong generalization, resistance to overfitting, and effectiveness in learning nonlinear patterns, CatBoost is considered a suitable and promising algorithm for predicting bond strength between steel reinforcement and geopolymer concrete.

### 2.3. Hyperparameter tuning using Bayesian optimization

Hyperparameter optimization was performed to improve the predictive performance of the developed machine learning models. In this study, Bayesian optimization was adopted to identify the optimal hyperparameter combination for each model. Compared with conventional search methods Bayesian optimization provides a more efficient strategy for exploring the hyperparameter space. It does so by building a probabilistic surrogate model of the objective function and sequentially selecting promising candidate solutions [24].

The collected dataset was first divided into two subsets. The training set comprised 80% of the total samples and was used for model development and hyperparameter optimization. The remaining 20% of the samples were reserved as an independent test set for the final performance evaluation. This data partitioning strategy was adopted to ensure that the generalization capability of the optimized models could be assessed on unseen data.

Within the training set, Bayesian optimization was coupled with a 5-fold cross-validation procedure. In each optimization iteration, the training data were randomly divided into five subsets of approximately equal size. Four subsets were used for model training, and the remaining subset was used for validation. This process was repeated five times so that each subset served once as the validation set. The average cross-validation performance across the five folds was used as the objective value to guide the optimization process [50].

For each machine learning algorithm, a predefined search space was specified for the corresponding hyperparameters. Bayesian optimization then iteratively evaluated candidate hyperparameter combinations and updated the surrogate model according to the observed cross-validation performance. An acquisition function was used to balance the exploration of unexplored regions and the exploitation of promising regions in the search space. Through this iterative procedure, the hyperparameter combination yielding the best average cross-validation performance was selected for each model. After optimization, the selected hyperparameters were used to retrain each model on the full training set. The optimized models were then evaluated on the independent test set. This final step was performed to examine the predictive accuracy and generalization ability of the developed models under unseen conditions.

### 2.4. Model evaluation metrics

The predictive performance of the ML models was assessed using four statistical indicators. The coefficient of determination (R²), root mean square error (RMSE), mean absolute percentage error (MAPE), and mean absolute error (MAE) were used. These metrics are commonly used in regression-based structural engineering studies. A higher R² value reflects stronger predictive capability. By contrast, lower values of RMSE, MAPE, and MAE indicate smaller prediction errors and better model accuracy. The mathematical formulations of these performance metrics are presented as follows:


$$\begin{array}{c} R^{\text{2}}=\text{1}-\frac{\sum_{i=\text{1}}^{n}{\left(y_{i}-{\hat{y}}_{i}\right)}^{\text{2}}}{\sum_{i=\text{1}}^{n}{\left(y_{i}-{\bar{y}}_{l}\right)}^{\text{2}}} \end{array}$$ (1)

$$\begin{array}{c} RMSE=\sqrt{\frac{\text{1}}{n}\sum_{i=\text{1}}^{n}{\left(y_{i}-{\hat{y}}_{i}\right)}^{\text{2}}} \end{array}$$ (2)

$$\begin{array}{c} MAPE=\frac{\text{1}}{n}\sum_{i=\text{1}}^{n}\frac{\left|y_{i}-{\hat{y}}_{i}\right|}{y_{i}}\times \text{ 100}\% \end{array}$$ (3)

$$\begin{array}{c} MAE=\frac{\text{1}}{n}\sum_{i=\text{1}}^{n}\left|y_{i}-{\hat{y}}_{i}\right| \end{array}$$ (4)

$$\begin{array}{c} \mu =\frac{\text{1}}{n}\sum_{i=\text{1}}^{n}\frac{T_{u\;pred}}{T_{u\;exp}} \end{array}$$ (5)

$$\begin{array}{c} \sigma =\sqrt{\frac{\text{1}}{n-\text{1}}\sum_{i=\text{1}}^{n}{\left(\frac{T_{u\;pred}}{T_{u\;exp}}-\mu \right)}^{\text{2}}\;} \end{array}$$ (6)

$$\begin{array}{c} COV=\frac{\sigma }{\mu }.\text{1}\text{0}\text{0}\% \end{array}$$ (7)

These evaluation metrics were used to assess the predictive performance of the developed models from different perspectives. Their combined use provides a more comprehensive evaluation of prediction accuracy, error magnitude, and model robustness. This approach also supports the identification of the most reliable model for bond strength prediction.

### 2.5. Shapley additive explanation (SHAP)

Machine learning models can achieve high predictive accuracy. However, their internal prediction process is often difficult to interpret. This limitation reduces model transparency and makes it difficult to understand how individual input variables influence the final output. In structural engineering applications, such interpretability is important because reliable prediction should be accompanied by a clear explanation of the governing factors. For this reason, Shapley Additive Explanations (SHAP) was employed in this study to interpret the developed machine learning model [25].

The SHAP value assigned to a variable represents its contribution to the prediction. This value is calculated by considering the marginal contribution of the variable across different combinations of input features. SHAP provides a consistent and quantitative measure of feature importance. It also allows both the magnitude and direction of each variable’s effect on the prediction to be examined. SHAP analysis was applied to the best-performing machine learning model to investigate the influence of input variables on the predicted bond strength between steel reinforcement and geopolymer concrete. Both global and local interpretability analyses were conducted. Global interpretation was performed using SHAP summary plots to identify the most influential variables affecting bond strength prediction. Local interpretation was further carried out to explain individual predictions by quantifying the contribution of each input parameter.

Using SHAP provides a clearer understanding of the relationships the model learns. It also improves the transparency and credibility of the prediction framework. The developed model can be interpreted not only in terms of predictive accuracy but also in terms of the relative importance and directional influence of the governing variables. This is useful for identifying the key factors that control the bond behavior between steel reinforcement and geopolymer concrete.

## 3. Model implementation and evaluation

### 3.1. Model training and test procedure

**Fig 4:**
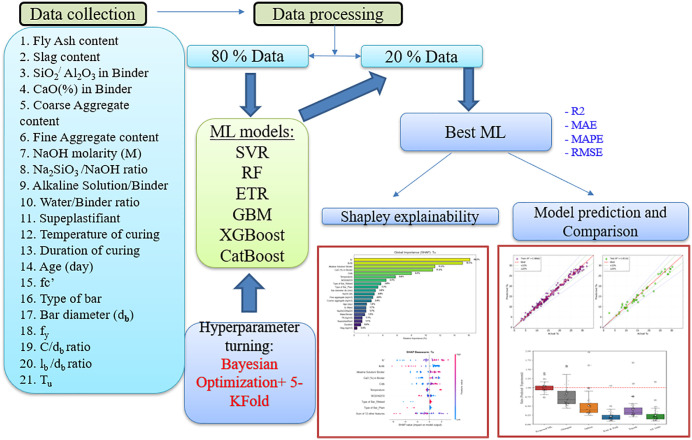
Model training and test procedure.

Fig 4 presents the workflow of the proposed machine learning framework for predicting the bond strength between steel reinforcement and geopolymer concrete. The workflow consists of database preparation, data preprocessing, model development, hyperparameter optimization, performance evaluation, and model interpretation. After preprocessing, the dataset is split into training and test sets at an 80/20 ratio. Six machine learning algorithms, including SVR, RF, ETR, GBM, XGBoost, and CatBoost, are implemented for model development. Bayesian optimization combined with cross-validation is then used to determine the optimal hyperparameters for each model. The predictive performance of the optimized models is assessed using statistical evaluation metrics. The best-performing model is finally interpreted using SHAP to quantify the influence of the input variables on bond strength prediction.

The computational framework was implemented in Python using established open-source libraries. The SVR, RF, ETR, and GBM models were implemented using Scikit-learn. The XGBoost and CatBoost models were implemented using the XGBoost and CatBoost Python libraries, respectively. SHAP-based model interpretation was performed using the SHAP library. Bayesian optimization was conducted using a Python-based optimization package. These libraries provided the core algorithmic implementations of the machine learning models. The original code developed by the authors was used to construct the modeling pipeline, including data preprocessing, one-hot encoding of categorical variables, train–test splitting, cross-validation, Bayesian optimization setup, model evaluation, residual analysis, repeated train–test validation, confidence interval estimation, empirical-model comparison, and visualization of results.

To further improve the statistical robustness of the model evaluation, a repeated train–test splitting procedure was additionally conducted. Instead of relying on a single 80/20 data partition, the dataset was repeatedly divided into training and testing subsets using different random seeds while maintaining the same training-to-testing ratio. For each repetition, the models were retrained and evaluated using the same optimized hyperparameter settings. The mean value and 95% confidence interval of each performance metric were then calculated to quantify the variability in model performance caused by data partitioning. This repeated evaluation strategy provides a more reliable assessment of model stability and reduces the risk that the reported performance depends on a particular random split. The 95% confidence interval was computed as:


$$\begin{array}{c} CI=\overset{―}{x}\pm t_{\text{0}.\text{9}\text{7}\text{5},n-\text{1}}\frac{s}{\sqrt{v}} \end{array}$$ (8)

where $\overset{―}{x}$ is the mean value of the evaluation metric over repeated runs, *s* is the standard deviation, and *n* is the number of repetitions and $t_{\text{0}.\text{9}\text{7}\text{5},n-\text{1}}$is the critical value of Student’s t-distribution.

### 3.2. Evaluation of model

The optimal hyperparameters obtained for each model are summarized in Table 2. These configurations correspond to the best results achieved during the Bayesian optimization process with 5 cross-validation. This optimization procedure enables efficient exploration of the hyperparameter space and improves model generalization. The selected hyperparameters also reflect the specific learning characteristics of each algorithm. These results highlight the importance of proper hyperparameter optimization for improving bond strength prediction.

Following the optimization stage, the predictive performance of all models is evaluated using several statistical indicators. The comparative results are presented in Table 3. A comprehensive assessment of the performance of RF, ERT, GBM, XGBoost and CatBoost were provide. The combined presentation of the optimized hyperparameters in Table 2 and the corresponding performance metrics in Table 3 enhances transparency and supports a fair comparison of the developed models in terms of accuracy, stability, and generalization.

**Table 3:** Average Predictive Performance of Models.

No	Model	Data Train				Data Test			
		R^2^	MAE	MAPE	RMSE	R^2^	MAE	MAPE	RMSE
1	SVR	0.971	0.755	5.320	1.288	0.923	1.540	15.733	2.079
2	RF	0.968	0.934	7.871	1.352	0.928	1.435	15.658	2.005
3	ETR	0.970	1.001	8.130	1.306	0.940	1.369	13.395	1.827
4	GBM	0.980	0.761	6.159	1.071	0.946	1.324	13.626	1.743
5	XGBoost	0.981	0.752	5.865	1.035	0.938	1.406	14.474	1.865
6	CatBoost	0.986	0.684	5.084	0.897	0.950	1.173	11.608	1.669

**Fig 5:**
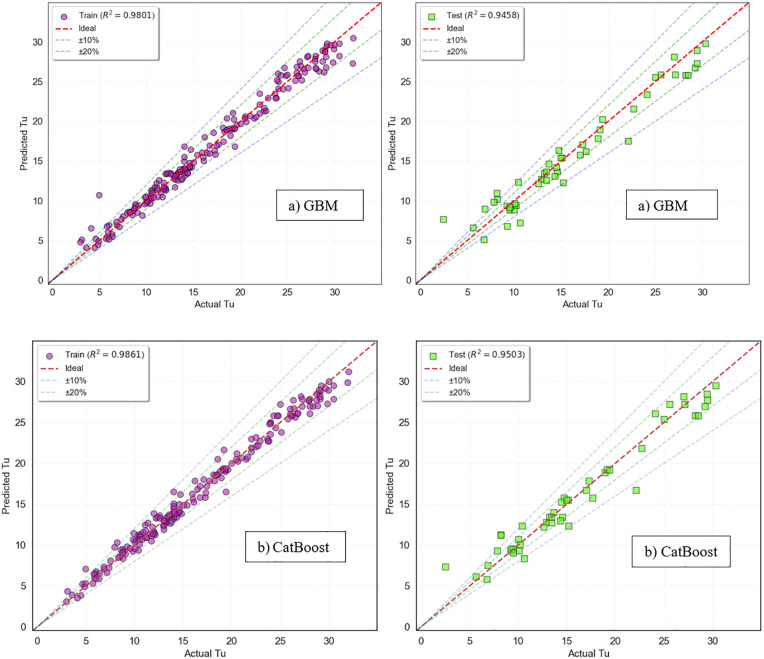
Relationship between actual and predicted values using the GBM and CatBoost models.

To evaluate the model performance on the training dataset using the K-fold technique, the data were partitioned into five subsets (5-K Fold). In each iteration, four folds were used for training while the remaining fold served as the validation set. The averaged results and the variability across the 5-K Fold are illustrated in the Fig 5.

After completing the model evaluation on the training portion of the dataset (constituting 80% of the total samples) and determining the optimal hyperparameters through the combined use of Bayesian optimization and 5-fold cross-validation, each optimized model was further subjected to validation on the independent test set comprising the remaining 20% of the data. This additional evaluation step was undertaken to examine the generalization capability of the models beyond the cross-validation framework and to ensure that the selected hyperparameters did not lead to overfitting on the training folds. The resulting performance metrics, including accuracy and error-based indicators, are systematically compiled in Table 3.

All models in this study showed strong fitting performance on the training dataset. The corresponding R² values ranged from 0.968 to 0.986. These values indicate that the selected input variables effectively capture the relationship between the governing parameters and bond strength. CatBoost outperformed the other models in terms of training performance with an R² of 0.986 and the lowest errors (MAE = 0.684, RMSE = 0.897, and MAPE = 5.084%). XGBoost and GBM are following, with training R² values of 0.981 and 0.980, respectively. The remaining models, including SVR, RF, and ETR, also showed satisfactory fitting capability, with R² values above 0.96.

The predictive performance was further examined using the independent testing dataset. The accuracy decreased slightly on unseen data. However, the overall results remained strong for all models. CatBoost again provided the best performance on the test set, with the highest R² of 0.950 and the lowest prediction errors (MAE = 1.173, RMSE = 1.669, and MAPE = 11.608%). GBM ranked second with a testing R² value of 0.946. ETR and XGBoost followed with R² values of 0.940 and 0.938, respectively. RF and SVR showed slightly lower test accuracies, with R² values of 0.928 and 0.923.

The ensemble boosting models, especially CatBoost and GBM, provided the most reliable predictive performance for bond strength prediction. These models achieved high accuracy on both the training and testing datasets. The results suggest that they offer good internal generalization capability within the range of the compiled experimental database. Among them, the CatBoost model consistently achieved the best performance across both training and testing datasets. Therefore, the CatBoost model was selected for the subsequent interpretability analysis. To quantitatively summarize the variability of model performance under repeated random data partitions, the mean values and 95% confidence intervals of the evaluation metrics are presented in Table 4.

**Table 4:** Repeated train–test validation results with 95% confidence intervals for the optimized machine learning models.

Model	(R2)	MAE	MAPE (%)	RMSE
SVR	0.923 ± 0.018	1.540 ± 0.210	15.733 ± 2.450	2.079 ± 0.280
RF	0.928 ± 0.016	1.435 ± 0.190	15.658 ± 2.260	2.005 ± 0.250
ETR	0.940 ± 0.014	1.369 ± 0.170	13.395 ± 1.980	1.827 ± 0.220
GBM	0.946 ± 0.012	1.324 ± 0.160	13.626 ± 1.850	1.743 ± 0.210
XGBoost	0.938 ± 0.015	1.406 ± 0.180	14.474 ± 2.050	1.865 ± 0.230
CatBoost	0.950 ± 0.011	1.173 ± 0.140	11.608 ± 1.620	1.669 ± 0.190

Note: The confidence intervals were estimated from repeated train–test evaluations using different random seeds. The values represent the mean performance ± 95% confidence interval.

As shown in Table 4, the repeated train–test validation provides a quantitative assessment of the sensitivity of model performance to random data partitioning. The CatBoost model achieved the best overall performance across the repeated evaluations, with high R2 and low error values. The relatively small variations in the evaluation metrics indicate that the predictive performance of the optimized CatBoost model remained stable under different training and testing subsets.

The repeated train–test evaluation further confirmed the stability of the proposed machine learning framework. Across repeated data partitions, the CatBoost model consistently achieved the best overall performance, with only small variations in R², MAE, MAPE, and RMSE. These results indicate that the predictive accuracy of the proposed model is not substantially affected by the random selection of training and testing samples. Therefore, the reported performance of the BO-CatBoost model can be considered statistically stable for internal validation within the range of the compiled experimental database.

The relatively small dataset size compared with the number of input variables may increase the risk of overfitting, especially for complex ensemble models. Therefore, several measures were adopted to improve model generalization. First, hyperparameter optimization was conducted using Bayesian optimization combined with 5-fold cross-validation on the training set. Second, the optimized models were evaluated using a hold-out testing set that was not involved in model training or hyperparameter tuning. Third, the training and testing performances were compared to identify possible overfitting. The relatively small performance gap between the training and testing results of the CatBoost model indicates that severe overfitting was not observed. Moreover, the repeated train–test evaluation further confirmed that the predictive performance remained stable across different random data partitions.

It should be noted that the hold-out testing set used in this study represents internal validation because it was randomly selected from the compiled database. Therefore, it should not be interpreted as a fully external validation dataset. Nevertheless, the combined use of 5-fold cross-validation, independent testing, and repeated train–test evaluation provides a more reliable assessment of model stability than a single data split. The generalization capability reported in this study should therefore be understood within the range of material, curing, and geometric conditions represented in the compiled experimental database.

Although the optimized CatBoost model achieved a high testing R^2^, this result should be interpreted with caution. The compiled database includes samples extracted from multiple literature sources, and some records may share similar mixture designs, testing protocols, or experimental conditions. Therefore, data dependency or hidden correlations among samples may partly influence the observed predictive performance. The reported accuracy should be regarded as internal predictive performance within the range of the compiled database rather than definitive evidence of external generalizability. Further validation using independent external datasets, study-wise validation, or newly generated experimental results is required to confirm the broader predictive capability of the proposed model.

### 3.3. ML models prediction performance of best machine learning model

As illustrated in Fig 5, the horizontal axis represents the experimentally measured bond strength (τ_*u*_), while the vertical axis denotes the predicted values obtained from two best model: GBM and CatBoost model for both the training and testing datasets. The red dashed line corresponds to the ideal prediction line (y = x), while the green and blue dashed lines indicate the ± 10% and ±20% error bands, respectively.

Fig 5 compares the predicted and experimental bond-strength values from the GBM and CatBoost models. In the training dataset, the predicted values from both models are closely distributed around the ideal line. CatBoost shows slightly better performance than GBM, as reflected by its higher R² value. A similar trend is observed in the testing dataset.

The GBM model achieved a coefficient of determination of R² = 0.9801, while the CatBoost model exhibited slightly higher accuracy with R² = 0.9861. In both cases, the majority of the training data points are concentrated within the ± 10% error band, demonstrating the strong capability of these ensemble learning models to capture the nonlinear relationships between the governing parameters and the bond strength response. A similarly strong predictive capability is observed for the testing dataset. The GBM model achieved R² = 0.9458, whereas the CatBoost model provided a slightly improved performance with R² = 0.9503, confirming its better generalization capability when applied to unseen data. Most of the testing data points are distributed within the ± 20% error bounds, with a considerable number still falling inside the ± 10% band, indicating that the predicted bond strengths remain in close agreement with the experimental values across a wide range of bond strength levels. Although the scatter widens slightly for unseen data, most points remain within ±20% error bounds, and many still fall within ±10% error bounds. These results confirm that both models provide reliable predictions, while CatBoost demonstrates slightly better accuracy and generalization performance.

**Fig 6:**
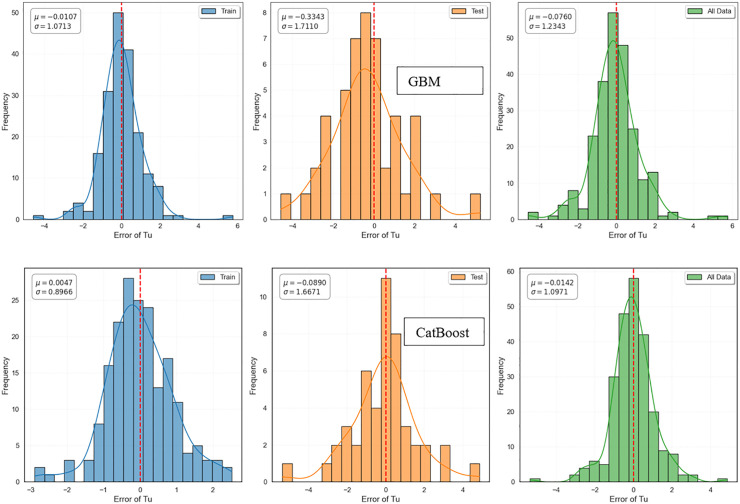
Distribution of prediction residuals for the GBM and CatBoost models in the training, testing, and entire datasets.

Fig 6 presents the residual distributions of the GBM and CatBoost models. For the CatBoost model, the residuals in the training dataset exhibit an approximately normal distribution centered very close to zero, with a mean error of μ = 0.0047 and a standard deviation of σ = 0.8966. The model predictions are essentially unbiased and that the majority of errors remain relatively small, confirming the strong fitting capability of the model for the training data. For the test dataset, the residual distribution also follows an approximately Gaussian pattern, with a mean of μ = −0.0890 and a standard deviation of σ = 1.6671. The slightly negative mean value indicates a slight tendency for the model to underestimate bond strength. However, the magnitude of this deviation remains small and does not indicate any systematic bias in predictions. The larger standard deviation compared with the training dataset reflects the expected increase in prediction uncertainty when the model is applied to previously unseen data. Across the entire dataset, the residuals are symmetrically distributed around zero, with μ = −0.0142 and σ = 1.0971, confirming the overall stability and robustness of the CatBoost model. Most residuals are concentrated within a relatively narrow range around zero, and no pronounced skewness or extreme outliers are observed. The results obtained from the predicted-versus-experimental plots and the residual analysis consistently demonstrate the strong predictive capability of the developed models. Both GBM and CatBoost show close agreement between predicted and observed bond strengths in the training and test datasets, indicating that the main patterns in the data were effectively captured. CatBoost exhibits slightly better performance in terms of prediction accuracy, error dispersion, and generalization to unseen data.

To further quantify the uncertainty associated with individual predictions, an approximate residual-based prediction interval was estimated for the optimized CatBoost model. The residual distribution of the testing dataset was used because it reflects the prediction error on unseen samples within the compiled database. Assuming that the testing residuals are approximately distributed around zero, the 95% prediction interval can be expressed as:


$$\begin{array}{c} {PI}_{\text{9}\text{5}\%}=\hat{y_{i}}\pm \text{1}.\text{9}\text{6}\sigma_{e} \end{array}$$ (9)

where $\hat{y_{i}}$ is the predicted bond strength and $\sigma_{e}$ is the standard deviation of the testing residuals. For the CatBoost model, the testing residual standard deviation was $\sigma_{e}$=1.6671. Therefore, the approximate 95% prediction interval was:


$$\begin{array}{c} {PI}_{\text{9}\text{5}\%}=\hat{y_{i}}\pm \text{3}.\text{2}\text{7} \end{array}$$ (10)

in the same unit as the target variable. This interval provides an approximate uncertainty range for individual predictions. It should be noted that this interval was derived from the residual distribution of the internal testing dataset. Therefore, it should be interpreted as an internal uncertainty estimate rather than an externally validated prediction interval.

As shown in Table 5, the testing dataset produced a wider prediction interval than the training dataset, which reflects the expected increase in uncertainty when the model is applied to unseen data. Nevertheless, the mean testing residual remained close to zero, indicating that the optimized CatBoost model did not show a strong systematic bias. The residual-based prediction interval provides a practical estimate of the reliability range of individual predictions within the compiled database.

**Table 5:** Residual-based prediction uncertainty of the optimized CatBoost model.

Dataset	Mean residual ($\mu_{e}$)	Residual standard deviation ($\sigma_{e}$)	Approximate 95% prediction interval
Training set	0.0047	0.8966	$\hat{y_{i}}\pm \text{1}.\text{7}\text{6}$
Testing set	−0.0890	1.6671	$\hat{y_{i}}\pm \text{3}.\text{2}\text{7}$
Entire dataset	−0.0142	1.0971	$\hat{y_{i}}\pm \text{2}.\text{1}\text{5}$

### 3.4. Comparison with existing empirical models

The empirical equations considered in this comparison were selected based on their direct applicability to the available experimental database. These models use variables that were consistently reported or could be derived from the collected studies, such as concrete compressive strength, bar diameter, concrete cover, and embedment length. International design standards, such as Eurocode, Model Code, Japanese, Chinese, and ACI provisions, were not included in the quantitative comparison because many of these provisions are primarily intended for development length or anchorage design rather than direct prediction of ultimate bond stress from pull-out tests. In addition, some code-based equations require design-specific parameters, including bar position, bond condition, confinement condition, transverse reinforcement, casting condition, and safety or modification factors. These parameters were not consistently available in the compiled database. Therefore, including such standards would require additional assumptions, which could undermine the fairness and consistency of the comparison. A broader evaluation involving international design provisions should be considered in future studies when more complete experimental information becomes available.

Table 6 summarizes several empirical models proposed in previous studies for estimating the bond strength between steel reinforcement and concrete. These analytical expressions typically relate bond strength to a limited number of parameters, most commonly the compressive strength of concrete (fc′), the concrete cover-to-bar diameter ratio (c/d_b_), and the embedment length-to-bar diameter ratio (lb/db). For instance, the model proposed by Orangun et al. [8] incorporates the effects of compressive strength, concrete cover, and embedment length, reflecting the influence of both material properties and geometric parameters on bond performance. Similarly, the design provisions in AS 3600 [51] estimate bond strength as a function of compressive strength and the concrete cover-to-bar diameter ratio.

**Table 6:** Empirical models proposed by different research.

No	Method	Equation	Reference
1	Orangun et al	$T_{u,cal}=\text{0}.\text{0}\text{8}\text{3}\sqrt{fc^{\prime }}\left[\text{1}.\text{2}+\text{3}\frac{c_{c}}{d_{b}}+\text{5}\text{0}\frac{d_{b}}{l_{b}}\right]$	[8]
2	AS 3600	$T_{u,cal}=\text{0}.\text{2}\text{6}\text{5}\sqrt{fc^{\prime }}\left[\frac{c_{c}}{d_{b}}+\text{0}.\text{5}\right]$	[51]
3	Topark-Ngram et al.	$T_{u,cal}=\text{2}.\text{1}\text{2}\sqrt{fc^{\prime }}$	[11]
4	Dahou et al	$T_{u,cal}=\text{3}.\text{8}\text{3}\sqrt{fc^{\prime }}$	[18]
5	Kim and Park	$T_{u,cal}=\sqrt{fc^{\prime }}\left[\text{2}.\text{0}\text{7}+\text{0}.\text{2}\frac{c_{c}}{d_{b}}+\text{4}.\text{1}\text{5}\frac{d_{b}}{l_{b}}\right]$	[19]

Other simplified models, such as those proposed by Topark-Ngram [11] et al. and Dahou [18], assume that bond strength is primarily governed by the compressive strength of concrete, resulting in equations that depend only on fc′. While these simplified relationships are convenient for engineering calculations, they may not fully capture the complex interactions among multiple parameters affecting the bond behavior. In contrast, the model proposed by Kim and Park [19] incorporates additional geometric variables, providing a more comprehensive representation of the bond mechanism.

**Fig 7:**
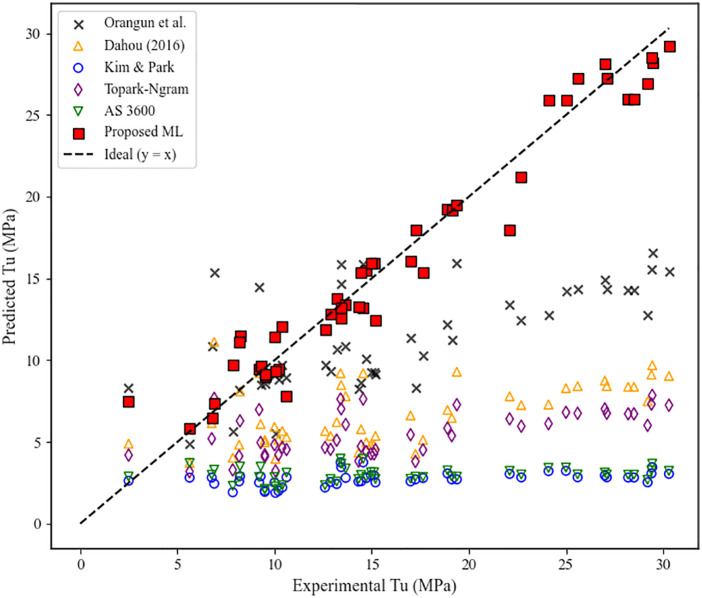
Comparison of the predictive performance of the proposed ML model and five empirical equations for bond strength.

Despite their simplicity and practical applicability, these empirical formulations are generally derived from limited experimental datasets and rely on predefined functional relationships between variables. As a result, their predictive capability may be restricted when applied to datasets with broader ranges of material compositions and geometric configurations, such as those encountered in geopolymer concrete systems. In the present study, the predictive performance of these traditional analytical equations is compared with that of an optimized CatBoost model, which is capable of capturing complex nonlinear relationships between input parameters and bond strength without requiring predefined mathematical forms (Fig 7).

**Fig 8:**
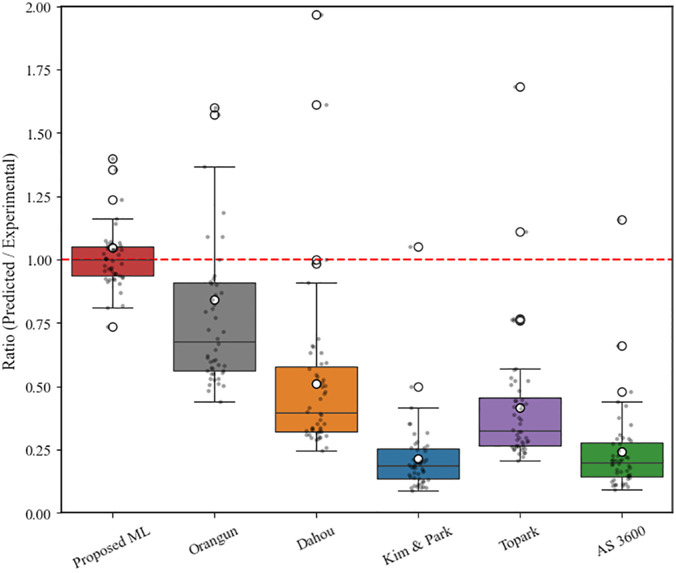
Box plot comparison of the proposed ML model and five empirical equations for bond strength.

The bond strength between steel reinforcement and geopolymer concrete predicted by the proposed machine learning (ML) model and five existing empirical equations is compared in Fig 8 and Table 7. The performance of each model is evaluated using statistical indicators, namely the mean ratio (μ), standard deviation (σ), and coefficient of variation (COV). In addition, a graphical comparison between predicted and experimentally measured bond strengths is presented to provide a comprehensive assessment of the predictive capability of each model.

**Table 7:** Statistical comparison of the predictive accuracy of the CatBoost model and existing empirical equations for estimating the bond strength between steel reinforcement and geopolymer concrete.

Models	Mean (µ)	Std (σ)	Cov (%)
BO-CatBoost	1.046	0.314	0.300
Orangun [8]	0.842	0.501	0.595
Dahou [18]	0.511	0.326	0.637
Kim &Park [19]	0.216	0.151	0.698
Topark-Ngram [11]	0.418	0.257	0.616
AS 3600 [51]	0.240	0.176	0.730

The statistical comparison of the predicted-to-experimental bond strength ratios for different models is summarized in Table 7. The proposed BO-CatBoost model demonstrates the best overall performance, with a mean ratio (µ) of 1.046, which is very close to the ideal value of 1.0. This indicates that the machine learning model provides an almost unbiased prediction of bond strength. In addition, the standard deviation (σ = 0.314) and coefficient of variation (COV = 0.300) are significantly lower than those of the empirical models. The proposed model produces more stable and consistent predictions.

The empirical models generally underestimate the bond strength. For instance, the Orangun model yields a mean ratio of 0.842, indicating a systematic underestimation of approximately 16%. The variability of this model is relatively high (σ = 0.501 and COV = 0.595), demonstrating a wide dispersion between predicted and experimental values. Similar trends are observed for the Dahou and Topark models, which produce mean ratios of 0.511 and 0.418, respectively.

The Kim & Park and AS 3600 models show even larger deviations from the experimental values, with mean ratios of 0.216 and 0.240, respectively. These very low mean values indicate substantial underestimation of bond strength. Additionally, their high coefficients of variation (COV = 0.698 and 0.730) reveal considerable prediction uncertainty. This discrepancy may be attributed to the fact that these empirical formulations were originally developed based on specific experimental conditions and material compositions that differ from those in the present database. The statistical indicators clearly demonstrate the superiority of the BO-CatBoost model over the conventional empirical equations. The machine learning approach not only achieves predictions closer to the experimental results but also significantly reduces prediction variability. Therefore, the proposed model provides a more reliable and robust tool for estimating the bond strength of geopolymer concrete reinforced with steel bars.

## 4. Model explain ability

### 4.1. SHAP-based analysis

Based on the comparative performance assessment, the CatBoost model was selected for SHAP-based interpretability analysis. This choice was primarily motivated by its consistently high predictive accuracy on the independent test dataset, accompanied by relatively low error metrics, indicating strong generalization capability. In addition, CatBoost demonstrated stable behavior across different data partitions, minimizing the risk of overfitting observed in several alternative models.

**Fig 9:**
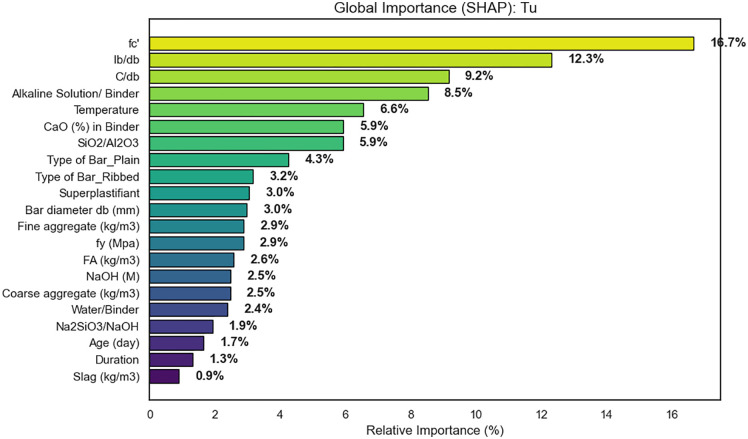
Relative importance of input features for bond between steel and geopolymer concrete.

The relative importance of the input variables for predicting the bond strength between steel reinforcement and geopolymer concrete (*T*_*u*_) was analyzed using SHAP global importance, as illustrated in Fig 9. The results reveal that both the mechanical properties of geopolymer concrete and the geometric characteristics of reinforcement play dominant roles in governing the bond behavior.

Among all variables, the compressive strength of geopolymer concrete (f′c) is identified as the most influential parameter, contributing 16.7% to the prediction of bond strength. This result is consistent with the fundamental mechanism of bond development, where higher concrete strength enhances adhesion, friction, and mechanical interlock between the steel surface and the surrounding matrix. Consequently, stronger geopolymer matrices provide greater resistance against bond slip and failure.

The embedment length-to-bar diameter ratio (l_b_/d_b_) represents the second most influential factor, with a relative importance of 12.3%. This parameter directly controls the available anchorage length and the distribution of bond stresses along the steel–concrete interface. A larger embedment length generally improves the efficiency of stress transfer and increases the ultimate bond capacity.

Another important geometric parameter is the cover-to-bar diameter ratio (c/d_b_), which contributes 9.2% to the prediction. Adequate concrete cover enhances confinement around the reinforcing bar, delaying splitting cracks and improving the bond resistance. The alkaline solution-to-binder ratio also exhibits significant influence (8.5%), highlighting the importance of mixture design in determining the microstructural characteristics of geopolymer concrete.

Parameters related to the geopolymer chemistry further contribute to bond behavior. In particular, curing temperature (6.6%), CaO content in the binder (5.9%), and the SiO_2_/Al_2_O_3_ ratio (5.9%) play important roles in controlling the geopolymerization process and the formation of reaction products. These chemical factors affect the development of gel phases such as N–A–S–H and C–(A)–S–H, which determine the density and mechanical strength of the geopolymer matrix. A denser microstructure improves the quality of the interfacial transition zone (ITZ) surrounding the reinforcing bar, thereby enhancing adhesion and bond performance.

Moderate contributions are also observed for parameters associated with reinforcement characteristics and mixture composition. The type of reinforcement bar, including plain bars (4.3%) and ribbed bars (3.2%), influences the mechanical interlocking mechanism at the steel–concrete interface. Deformed (ribbed) bars generally provide stronger interlock compared with plain bars, which improves bond resistance. Other variables such as superplasticizer dosage (3.0%), bar diameter (3.0%), fine aggregate content (2.9%), and steel yield strength (2.9%) show moderate but noticeable effects on bond behavior.

Finally, several mixture parameters exhibit relatively smaller contributions, including fly ash content (2.6%), NaOH concentration (2.5%), coarse aggregate content (2.5%), water-to-binder ratio (2.4%), Na₂SiO₃/NaOH ratio (1.9%), curing age (1.7%), curing duration (1.3%), and slag content (0.9%). Although their individual impacts are limited, these variables collectively influence the geopolymer matrix formation and may indirectly affect bond performance.

The SHAP analysis demonstrates that the bond strength of steel reinforcement in geopolymer concrete is governed by a complex interaction between concrete mechanical properties, reinforcement geometry, and geopolymer mixture chemistry. While compressive strength and anchorage geometry dominate the macroscopic bond mechanism, the chemical composition of the geopolymer binder plays a crucial role in controlling the microstructure and the quality of the steel–concrete interface. These findings provide valuable insights for optimizing both mixture design and reinforcement detailing to enhance the bond performance of geopolymer concrete structures.

### 4.2. Feature dependency analysis

**Fig 10:**
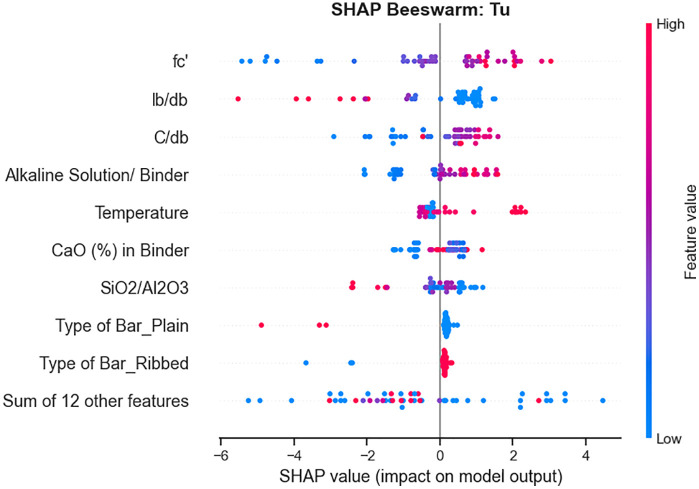
SHAP Beeswarm Plot.

The SHAP beeswarm plot shown in Fig 10 was used to further examine the influence of individual variables on the predicted bond strength. Each point represents an individual observation, while the color gradient indicates the feature value ranging from low (blue) to high (red). Positive SHAP values indicate that the variable increases the predicted bond strength, whereas negative values suggest a reduction in the predicted output.

Consistent with the global SHAP importance results, the compressive strength of geopolymer concrete (fc’) exhibits the strongest influence on the predicted bond strength. In the beeswarm distribution, higher values of fc’ (red points) are predominantly located on the positive SHAP side, indicating that increasing compressive strength generally leads to higher predicted bond capacity. This behavior is consistent with fundamental bond mechanics, where a stronger concrete matrix enhances adhesion, friction, and mechanical interlocking at the steel–concrete interface.

The embedment length-to-bar diameter ratio (l_b_/d_b_) also shows a strong positive influence on the model output. Higher values of l_b_/d_b_ are mainly associated with positive SHAP values, confirming that a larger embedment length improves anchorage capacity and facilitates more efficient stress transfer along the steel–concrete interface. Similarly, the cover-to-bar diameter ratio (c/d_b_) demonstrates a noticeable positive contribution, indicating that greater concrete cover enhances confinement around the reinforcement and delays splitting failure, thereby improving bond resistance.

For mixture-related parameters, the alkaline solution-to-binder ratio and curing temperature exhibit clear positive trends in the SHAP distribution. Higher values of these parameters generally correspond to positive SHAP values. This result indicates that enhanced geopolymerization conditions and improved reaction kinetics favor the development of a denser matrix and a stronger interfacial transition zone (ITZ). The CaO content in the binder and the SiO₂/Al₂O₃ ratio also exert moderate influences on the predicted bond strength. Their effects reflect the important role of binder chemistry in governing the chemical composition and microstructural evolution of the geopolymer matrix.

In contrast, some variables such as reinforcement type and aggregate contents exhibit more scattered SHAP distributions around zero. This dispersion indicates that their effects on bond strength are relatively moderate and may interact with other parameters in a nonlinear manner. The broader spread of SHAP values observed for several variables highlights the capability of the CatBoost model to capture complex interactions among mixture composition, reinforcement geometry, and curing conditions when predicting the bond strength between steel reinforcement and geopolymer concrete.

### 4.3. ICE and PDP

**Fig 11:**
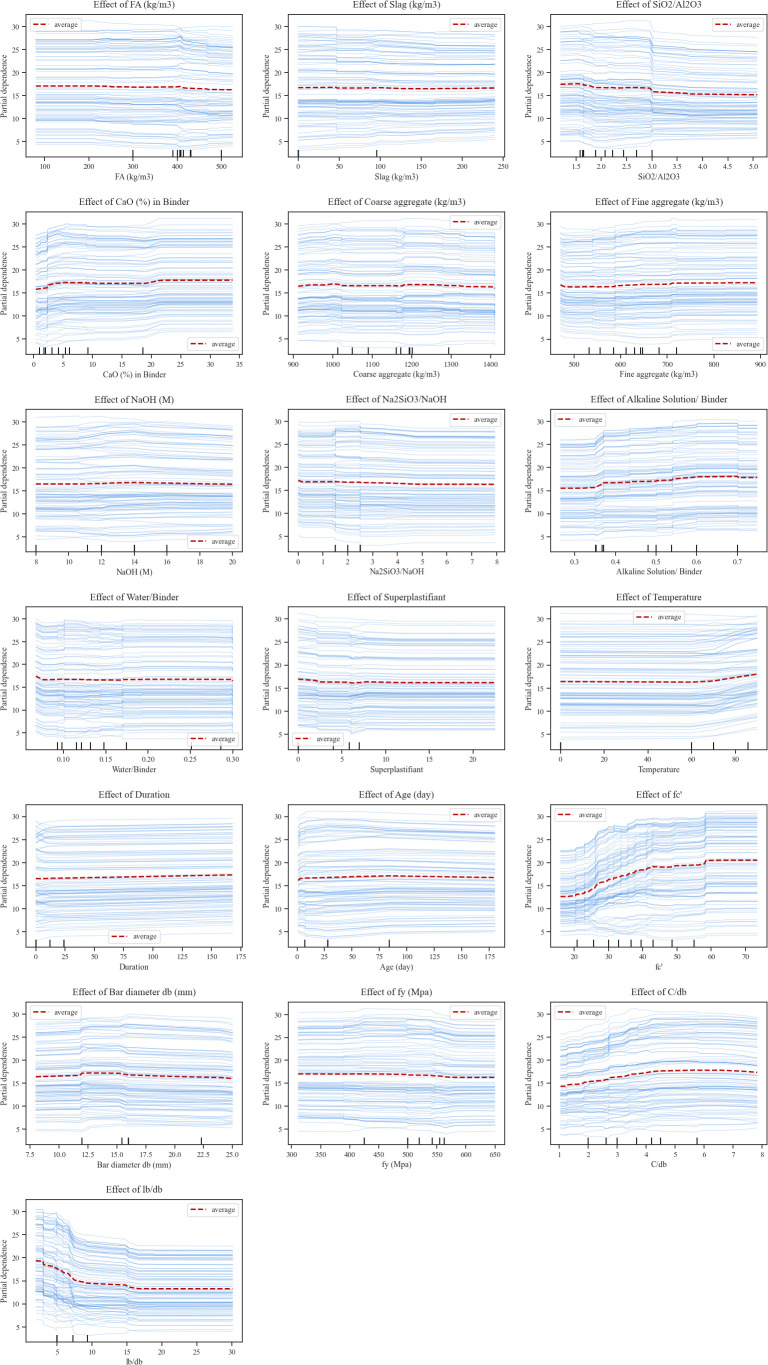
ICE (Individual Conditional Expectation) and PDP – (Partial Dependence Plot) analyses.

ICE–PDP (Individual Conditional Expectation and Partial Dependence) plots were further employed to examine the effects of individual variables on the predicted bond strength (Fig 11). The thin blue lines show the prediction trends for individual samples. The red dashed line indicates the average partial dependence. It represents the overall trend of each variable with respect to the predicted bond strength.

The results clearly indicate that the compressive strength of geopolymer concrete (fc’) exerts a strong positive influence on the predicted bond strength. As the compressive strength increases, the average predicted bond strength (τ_u_) increases noticeably. This behavior is consistent with classical bond mechanics, where a stronger concrete matrix enhances adhesion, friction, and mechanical interlocking between the steel reinforcement and the surrounding concrete. The geometric parameter representing confinement conditions, namely the cover-to-bar diameter ratio (c/d_b_), also exhibits a positive relationship with the predicted bond strength. As c/d_b_ increases, the PDP curve shows a gradual increase followed by a slight stabilization at higher values, indicating that increased concrete cover improves confinement and delays splitting cracks around the reinforcing bar. Conversely, the embedment length-to-bar diameter ratio (l_b_/d_b_) demonstrates a decreasing trend in the partial dependence curve. The predicted bond strength decreases sharply at lower values of l_b_/d_b_ and then gradually stabilizes, suggesting that once sufficient embedment length is provided, further increases contribute less effectively to improving the bond capacity.

Several parameters related to geopolymer mixture composition also exhibit noticeable effects. The alkaline solution-to-binder ratio shows a moderate increase, indicating that a higher alkaline activator content enhances geopolymerization and improves matrix densification. Similarly, curing temperature positively influences the predicted bond strength, reflecting its role in accelerating reaction kinetics and promoting the formation of geopolymer gels. The CaO content in the binder also demonstrates a mild positive trend before stabilizing, suggesting that moderate calcium content contributes to the formation of additional reaction products that improve matrix strength and the quality of the interfacial transition zone (ITZ).

Other mixture parameters such as fly ash content, slag content, NaOH molarity, Na_2_SiO_3_/NaOH ratio, water-to-binder ratio, and aggregate contents exhibit relatively mild variations in the PDP curves. These variables generally produce small fluctuations in the predicted bond strength, suggesting that their effects are secondary to the dominant mechanical and geometric parameters. Slight nonlinear responses can still be observed for certain variables, particularly the SiO_2_/Al_2_O_3_ ratio and aggregate content, suggesting that complex interactions may exist within the geopolymer mixture.

The ICE curves further reveal variability among individual samples, suggesting that the influence of each variable may differ across specific combinations of input parameters. This spread of individual trajectories highlights the nonlinear interactions captured by the machine learning model. Nevertheless, the overall trends represented by the PDP curves remain physically meaningful and consistent with established bond behavior mechanisms.

The ICE–PDP analysis enhances the interpretability of the machine learning model by illustrating how variations in individual variables influence the predicted bond strength. The results confirm that the bond behavior of steel reinforcement in geopolymer concrete is primarily governed by concrete compressive strength, reinforcement geometry, and confinement conditions, while geopolymer mixture chemistry and curing parameters contribute secondary but still meaningful effects on the overall bond performance.

## 5. Limitation and future recommendations

Despite the promising predictive capability achieved by the proposed machine learning models, several limitations should be acknowledged. The primary limitation relates to the size and diversity of the experimental dataset used in this study. Although the compiled database integrates bond test results from multiple published studies, the overall number of available experiments on the bond behavior between steel reinforcement and geopolymer concrete remains relatively limited compared with the extensive datasets available for conventional Portland cement concrete. Consequently, the dataset does not fully capture the wide variability of parameters that may influence bond performance in practical applications.

The relatively small dataset size in relation to the number of input variables may increase the risk of overfitting, especially for complex ensemble models. Several measures were adopted to reduce this risk. These measures included 5-fold cross-validation, hold-out testing, Bayesian optimization, repeated train–test evaluation, and comparison between training and testing performance. Nevertheless, the model may still be sensitive to sparsely represented regions of the input space. Important factors such as bar diameter, embedment length, concrete cover, geopolymer mix composition, curing regimes, and surface characteristics of reinforcement bars are not uniformly represented across the collected data. As a result, the prediction results should be interpreted within the range of the compiled experimental database.

Another limitation stems from the heterogeneity of the experimental data collected from different sources. The bond test results were obtained from independent experimental programs that may involve variations in specimen preparation, geopolymer formulations, curing conditions, loading procedures, and measurement techniques. Although careful data screening and preprocessing were performed to minimize inconsistencies, the lack of standardized experimental protocols for geopolymer concrete bond tests may introduce unavoidable uncertainties into the compiled dataset. Such variability could influence the learning process of data-driven models and affect their predictive robustness.

A further limitation is the absence of a fully independent external validation dataset. The hold-out testing set and repeated train–test evaluations used in this study were still based on the compiled database. Therefore, these procedures should be regarded as internal validation rather than true external validation. The reported predictive performance and generalization capability should therefore be understood within the parameter ranges covered by the present database. Future studies should validate the proposed framework using newly generated experimental results or independent datasets that are not involved in model development.

Future research should therefore prioritize expanding the available experimental database to include a broader range of geopolymer concrete mixtures, reinforcement geometries, and bond test configurations. Establishing a standardized and open-access database dedicated to the bond behavior between steel reinforcement and geopolymer concrete would greatly benefit the research community. A collaborative data-sharing platform would facilitate consistent reporting of experimental parameters and accelerate the accumulation of reliable datasets, thereby improving the generalization capability of machine learning models.

In addition, advanced data-driven techniques may be explored to enhance model reliability. Synthetic data generation methods, probabilistic sampling strategies, and generative modeling approaches could be applied to mitigate data imbalance and enrich sparsely represented regions of the parameter space. Furthermore, integrating metaheuristic optimization algorithms such as Particle Swarm Optimization (PSO) [52], Grey Wolf Optimizer [53] into the model development framework may further improve hyperparameter tuning and predictive accuracy. With the continued growth of experimental data and methodological advancements, the proposed machine learning framework can be progressively refined and extended to more complex reinforcement systems and geopolymer concrete formulations in future studies.

## 6. Conclusions

This study developed a Bayesian-optimized and interpretable machine learning framework for predicting the bond strength between steel reinforcement and geopolymer concrete. The main conclusions drawn from this study are as follows:

- Six machine learning models were evaluated, including SVR, RF, ETR, GBM, XGBoost, and CatBoost. With testing R^2^ values ranging from 0.923 to 0.950, all models demonstrated strong predictive accuracy. CatBoost outperforms another model. The optimized CatBoost model produced the highest testing accuracy, with R² = 0.950, MAE = 1.173, MAPE = 11.608%, and RMSE = 1.669.
- The comparison with existing empirical equations confirmed the advantage of the proposed data-driven approach. The optimized CatBoost model showed a mean ratio close to unity and a lower coefficient of variation, whereas the empirical equations generally exhibited larger variability and systematic underestimation.
- The SHAP analysis identified compressive strength, the embedment length-to-bar diameter ratio, and the cover-to-bar diameter ratio as the most influential parameters affecting bond strength prediction. Several mixture-related variables, including the alkaline solution-to-binder ratio, curing temperature, CaO content, and the SiO_2_/Al_2_O_3_ ratio, also showed noticeable contributions.

The proposed framework provides an accurate and interpretable tool for bond strength prediction in steel-reinforced geopolymer concrete. This approach can support bond assessment and reduce reliance on extensive experimental testing, while future studies should expand the database and incorporate additional material- and microstructure-related information to improve model robustness further.

## Supporting information

S1 Data SI data 1. (CSV)
